# MimoPro: a more efficient Web-based tool for epitope prediction using phage display libraries

**DOI:** 10.1186/1471-2105-12-199

**Published:** 2011-05-25

**Authors:** Wen Han Chen, Ping Ping Sun, Yang Lu, William W Guo, Yan Xin Huang, Zhi Qiang Ma

**Affiliations:** 1 School of Computer Science and Information Technology, Northeast Normal University, Changchun 130024, P.R. China; 2 Faculty of Chemistry, Northeast Normal University, Changchun 130024, P.R. China; 3 School of Information and Communication Technology, Central Queensland University, North Rockhampton QLD 4702, Australia; 4 National Engineering Laboratory for Druggable Gene and Protein Screening, Northeast Normal University, Changchun 130024, P.R. China

## Abstract

**Background:**

A B-cell epitope is a group of residues on the surface of an antigen which stimulates humoral responses. Locating these epitopes on antigens is important for the purpose of effective vaccine design. In recent years, mapping affinity-selected peptides screened from a random phage display library to the native epitope has become popular in epitope prediction. These peptides, also known as mimotopes, share the similar structure and function with the corresponding native epitopes. Great effort has been made in using this similarity between such mimotopes and native epitopes in prediction, which has resulted in better outcomes than statistics-based methods can. However, it cannot maintain a high degree of satisfaction in various circumstances.

**Results:**

In this study, we propose a new method that maps a group of mimotopes back to a source antigen so as to locate the interacting epitope on the antigen. The core of this method is a searching algorithm that is incorporated with both dynamic programming (DP) and branch and bound (BB) optimization and operated on a series of overlapping patches on the surface of a protein. These patches are then transformed to a number of graphs using an adaptable distance threshold (ADT) regulated by an appropriate compactness factor (*CF*), a novel parameter proposed in this study. Compared with both Pep-3D-Search and PepSurf, two leading graph-based search tools, on average from the results of 18 test cases, MimoPro, the Web-based implementation of our proposed method, performed better in sensitivity, precision, and Matthews correlation coefficient (*MCC*) than both did in epitope prediction. In addition, MimoPro is significantly faster than both Pep-3D-Search and PepSurf in processing.

**Conclusions:**

Our search algorithm designed for processing well constructed graphs using an ADT regulated by *CF *is more sensitive and significantly faster than other graph-based approaches in epitope prediction. MimoPro is a viable alternative to both PepSurf and Pep-3D-Search for epitope prediction in the same kind, and freely accessible through the MimoPro server located at http://informatics.nenu.edu.cn/MimoPro.

## Background

In humoral immunity, a pathogenic antigen is recognized by an antibody or B-cell receptor (BCR) through some regions on the outer surface of the antigen that are commonly known as the B-cell epitope. Since humoral responses are induced by epitopes on the surface of an antigen, rather than the whole antigen, it is important to locate these epitopes for the purpose of effective vaccine design. The most reliable methods for identification of epitopes are X-ray crystallography and NMR techniques, but they are time-consuming and expensive. Although using computational methods to predict epitopes is faster and cheaper, people still hold some doubts on the reliability of such techniques, compared with those experimental methods. Therefore incorporating experimental and computational methods in epitope prediction, such that epitope candidates are selected by computational methods prior to laboratory experiments, can lead to both significantly reducing the experimental cost and substantially accelerating the process of identification.

A continuous B-cell epitope is composed of residues in a single sequence of peptides whereas a discontinuous B-cell is constituted of multiple segments of amino acids. It has been reported that more than 90% of B-cell epitopes are discontinuous B-cell epitopes [1]. Early computational methods for epitope prediction were mostly focused on finding linear B-cell epitopes using different propensity scales and epitopic motifs which are derived from peptide sequences [2-6].

Theoretically, the 3D structure of a protein can provide more information than the amino acid sequence can. Therefore a good understanding of such 3D structure should lead to significant improvement in epitope prediction. CEP proposed in 2005 [7] and DiscoTope proposed in 2006 [8] are good examples of using such 3D information in epitope prediction. Recently proposed methods [9,10] have demonstrated further improvement on the performance in epitope prediction. Despite these achievements, epitope prediction is still a challenging task because epitopes are context dependent [11]. This means that the surface of an antigen is full of potential epitopes but the active epitopes depend on the antibody binding to the antigen in certain interactions.

Predicting B-cell epitopes using a phage display library takes the following procedure in general. Firstly, random peptides are displayed on the surface of filamentous phages. These random peptides which bind to a monoclonal antibody with a certain degree of affinity are then screened and amplified. This process is repeated, and with increase in number of iterations the resultant peptides become fewer but with a higher affinity. These affinity-selected peptides are also called mimotopes that have the similar functionality to and a high sequential similarity with the native epitope [12,13]. These features imply that certain key binding motifs and physicochemical preferences exist during interactions. Because mimotopes derived from the phage display technique share a common motif, mapping these mimotopes back to the source antigen can help finding the genuine epitope more accurately.

In recent years, trials have been made on mimotope mapping and several software packages have been developed accordingly. These tools can be classified into two major categories. One is to map mimotopes to the overlapping patches on the surface of an antigen using statistical features of mimotopes, such as physicochemical properties. Examples of this category include MIMOX [14], 3DEX [15], SiteLight [16], and Mapitope [17]. The other is to map mimotopes back to the genuine epitopes through aligning methods, such as FINDMAP [18], PepSurf [19], and Pep-3D-Search [20].

Mimotope mapping was firstly formulated as a graph searching problem by Mayrose et al. [19], and its objective was to find a group of simple paths on a graph generated from the residues on the surface of an antigen with the best match to the query mimotopes. It has been proven that finding a simple path on a graph is a *NP*-complete problem [18,21]. To make this intensive computation relatively efficient, PepSurf utilized a stochastic-based color-coding method [22] whereas Pep-3D-Search adopted an ant colony optimization (ACO) algorithm [23].

Finding a simple path on a graph is computationally intractable for any large-scale searching problem, but satisfactory performance can be achieved if the problem is kept in a small search space. In this paper, we propose a patch-based graph searching method that searches through all nodes on a regulated graph that has a confined small number of nodes. On each single patch, a complete search is conducted to guarantee the best alignment for each mimotope sequence. Dynamic programming (DP) [24] and branch & bound (BB) [25] method are also adopted to both avoid repetition in searching and further narrow the search space during processing. Furthermore, compared with previous work, we introduce an ADT to delineate a small area so that all amino acids within that area are regarded as the neighbor amino acids. This ADT is determined by a compactness factor (*CF*) which is modified from clustering coefficient firstly proposed in [9]. Adoption of such an ADT should better reflect the structural differences of various antigen surfaces. The results from a validation data set have confirmed that our method is more sensitive and faster in epitope prediction.

Our algorithm has been implemented as a Web-based tool named as MimoPro (Mimotope Projection) for public access, through which users around the world are able to carry out further validation and new applications. The MimoPro server is located at http://informatics.nenu.edu.cn/MimoPro.

## Methods and Implementation

### Overview of the proposed method

Our method aims at mapping a number of mimotopes back to the surface of an antigen so as to locate the interacting epitope on the antigen. Therefore, the required input includes both the X-ray crystal structure of a source antigen stored in a protein data bank (PDB) [26] and mimotopes screened from phage display experiments. The output of this mapping is a candidate epitope through the paths aligned to corresponding mimotopes.

**Figure 1:**
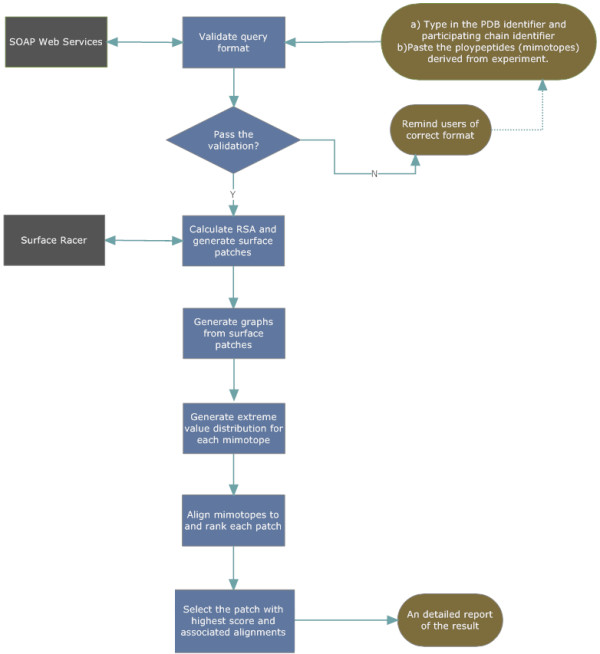
**Basic process of MimoPro**. Blocks on the right denote both the start of a request submitted by a user and the end of a request when the processed results are sent to the user by the specified email. The middle part is the flowchart of MimoPro process, which consists of six sequential functional blocks. Blocks on the left denote external services, with which MimoPro interacts during processing.

Initially, the antigen surface is divided into some overlapping patches with a radius of 15 Å and each patch is centered at atom ***C_β_***of a surface residue. Secondly, surface patches are further transformed to graphs bounded by neighboring amino acids that are determined using an ADT adjusted by *CF*. Mapping then becomes finding the best matched path for each mimotope in each graph. Since paths may have different lengths, to assess the similarity between a path and a mimotope sequence and to give consensus scores to paths with different lengths, we employ a statistical scoring norm called *P*-value that is derived from the extreme value distribution (EVD) for each mimotope [19]. Afterwards a patch-based searching algorithm is utilized to find the best alignment for each mimotope sequence in each graph. The similarity between a path and the corresponding mimotope is rated by *P*-value, and the patch with the highest score is retained as a potential candidate for the native epitope. This process is illustrated in Figure 1.

### Generation of surface patches

Since an epitope is a cluster of residues distributed on the surface of an antigen, the surface residues are firstly extracted from an antigen structure using solvent exposure [27]. Solvent exposure is commonly measured by solvent accessible surface area (ASA) which was firstly defined by Lee & Richards [28]. ASA of a residue is calculated as the sum of exposed areas of atoms using a 'rolling ball' algorithm developed by Shrake & Rupley [29]. This algorithm has been implemented by different researchers for academic use [30,31]. We choose Surface Racer 4.0 [31] to calculate ASA using a probe with a radius of 1.4 Å.

Similar to prior studies, we use relative solvent accessibility (RSA) of a residue as the surface residue. RSA of a residue is defined as ASA of a residue in proportion to the maximum exposed area of the same type. The maximum exposed area is measured as the exposed area of any type of amino acid in an ALA-X-ALA tripeptide [32]. In this study, ASA of each residue is firstly calculated with a probe radius of 1.4 Å; the sum of ASA of all member atoms is then calculated automatically; finally, any residue with RSA larger than a predefined threshold of 0.05 Å^2 ^is determined as a surface residue.

We choose a patch with a radius of 15 Å so that most epitopes can be encompassed in such a patch [33]. Because most patches contain no more than 50 residues, and most epitopes are distributed in loose and/or protruding regions of an antigen surface [8], patches containing more than 50 residues are precluded from consideration. To determine if a residue falls into a patch, position of the residue must be specified first. Commonly, a residue can be positioned at ***C_α_***, ***C_β _***or AHA atom of an amino acid [20]. Since an antigen interacts with corresponding antibody through the side chain, we think that the distance between two ***C_β _***atoms may better represent the spatial closeness of two neighboring residues.

### Compactness factor and generation of graphs

In graph theory, a graph is defined as ***G ***= (***V***, ***E***) where ***V ***denotes a collection of vertices and ***E ***denotes a collection of edges between any two vertices. To generate a graph from a surface patch, each residue on the surface patch is regarded as a vertex and the connection between a pair of vertices that is smaller than a predefined distance threshold is considered to be an edge. This has been realized using a fixed distance threshold (FDT) in all previous studies reported so far. The drawback of using an FDT is that the selection of a proper distance threshold is both difficult and irrational to some extent. This is mainly because different proteins have different structures, and even a single protein contains many different regions. Logically these different regions vary largely in spatial compactness. Therefore, a rationalized distance threshold should be adjustable so that a longer distance is used in the loose regions of an antigen to include more useful connections whereas a shorter distance is adopted in the dense regions to preclude some insignificant connections.

In this study, we use an ADT that is changeable in different regions of an antigen so that all resultant graphs share a uniform compactness. To estimate the compactness of a graph, we introduce a new parameter named as compactness factor (*CF*) that is modified from the clustering coefficient proposed in [9]. The compactness of a graph with different number of vertices is estimated by the observed number of edges in proportion to the expected number of edges, which is formulated as:(1)

where *e *denotes the observed number of edges under a specified distance threshold; *a *is an empirical constant set to 4; *k *is the number of vertices in the graph. The production of *a*×*k *is the expected number of edges.

Originally the expected number of edges is estimated by *k*(*k *- 1)/2, which is the maximum number of edges in a graph of *k *vertices [9]. With increase in number of vertices, the expected number of edges grows much faster than the observed number of edges does. Since each graph is transformed from a small patch that contains a limited number of residues (or vertices) and a residue only interacts with its neighbors in our problem, the expected number of edges is estimated to grow linearly with the number of vertices in the graph.

**Figure 2:**
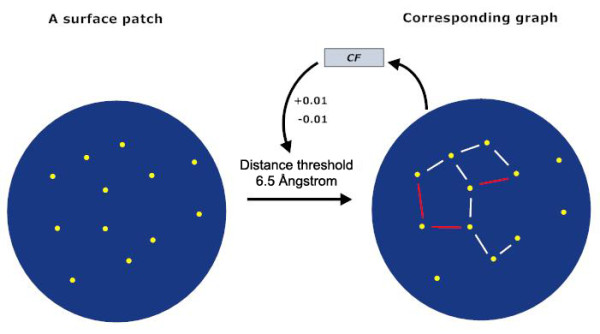
**Generation of a graph from a surface patch**. Dots in the left circle in yellow are residues inside a surface patch. Generation of a graph starts at setting a default distance threshold of 6.5 Å. A graph can be generated from the surface patch by specifying the connection between pairs of vertices (residues) within the distance threshold. Such a graph is shown in the right circle where edges are colored in white. The ***CF ***value can be calculated from this graph. If the value is smaller (or greater) than the bottom (or top) bound of a pre-specified ***CF ***value, the distance threshold is increased (or decreased) by 0.01 and the corresponding graph is generated. New edges colored in red are added into the previous graph. This process can be repeated until the ***CF ***value falls into the specified range.

Figure 2 shows an example of graph generation guided by *CF*. Normalization of compactness for all graphs regulated by *CF *also makes searching simpler and faster compared with previous methods.

### Mimotope mapping using dynamic programming

The patch-based complete search through dynamic programming (DP) [24] can be used to locate the active epitope on an antigen surface using a mimotope library. Since every mimotope may contain some information about the active epitope, all mimotopes are equally treated as query sequences and aligned to the best matched paths in each graph. Mapping a single mimotope sequence in a graph includes two tasks: scanning all potential paths in a graph, and assessing the similarity between each path and the mimotope sequence. A local alignment approach, including operations of replacement, insertion, deletion, and no-gap penalty at both ends of an alignment, is adopted to rank a path in a graph.

In every single alignment, a mimotope of length *k *is treated as a query denoted by *Q*(*k*) = (*q*_1_,...,*q_k_*), where *q_i _*denotes the *i *th amino acid in the query sequence (mimotope). A graph can be treated as a collection of simple paths, and each path is represented by *P*(*k*) = (*p*_1_,...,*p_k_*), where *p_i _*stands for the *i *th vertex (residue) in a graph. Further, we employ a substitution matrix to specify the penalty of replacement for a pair of amino acids: one being from a query sequence, and the other being aligned to the antigen surface. The score for the alignment between a path and a query sequence can be calculated by:(2)

where *W*(*Q*(*k*)*, P*(*k*)) denotes the score for the alignment between path *P*(*k*) and query mimotope sequence *Q*(*k*); *h*(*q_i_, p_i_*) denotes the penalty for a vertex *p_i _*in path *P*(*k*) with respect to an amino acid *q_i _*in mimotope sequence *Q*(*k*).

Most mimotopes from the phage display experiments contain no more than 15 amino acids. Since the compactness of any graph is controlled by a *CF *of 0.73 ± 0.06 in this study and the size of each graph is restricted to 50 vertices at most, we can make sure that the searching space is small enough to perform a complete search efficiently. Theoretically the search algorithm should explore all the potential paths in a graph and identify the path that best matches the mimotope sequence. Dynamic programming (DP) can reduce the number of repeating searches and prune some insignificant paths encountered in the traditional search algorithm.

A query sequence *Q*(*k*) (mimotope) is divided into *k *shorter strings. Each string starts at the first amino acid and ends at the *i*th amino acid denoted as *Q*(*i*). During the processing, each query string *Q*(*i*) is aligned to a graph iteratively and incrementally. A simple path *P*(*m, S*) which matches the query string *Q*(*i*) is represented by the last vertex *m *it ends at and a set of visited vertices (*S*). Increment of path *P*(*m, S*) from the *i*th step to the (*i*+1)th step is achieved by adding either vertex *j *that connects to *m *or a gap, whichever having a smaller penalty. Paths that end at the same vertex and share the same set of visited vertices but with different permutations are repeated paths. Among these repeated paths, the path with the highest score is retained only and other paths are regarded less significant.

The DP process starts at scoring each vertex in a graph from the first amino acid of a mimotope query sequence, which is also the shortest query string. A path ending at the current vertex is created by adding the current vertex into *S*, and the score for this newly generated path takes the highest among the penalty for a gap, the penalty for a replacement, or zero. The score should not be below zero since we place no penalty at both ends of an alignment.

As a query grows from *Q*(*i*) to *Q*(*i*+1), a new path for *Q*(*i*+1) is generated through: 1) calculating the penalty for amino acid replacement; 2) comparing the penalty for the replacement with the penalty for a gap, and adding whichever the higher to the path at spot *i*; 3) a matched path for query string *Q*(*i*+1) being generated by appending vertex *j *(or gap if no vertex) to a matched path for *Q*(*i*) resulted from the *i*th step ending at vertex *m *(*m *and *j *are connected vertices). When the iteration finally ends at string *Q*(*k*), the best matched path in a graph for *Q*(*k*) is obtained by scanning the best alignments ended at each vertex. Such iteration should identity all possible paths that have the potential to be the best alignment for query *Q*(*k*).

The DP process can be summarized by the following recursion:(3)(4)

*W*[*Q*(*i*)*,P*(*j, S*)] stands for the score of the alignment between the query string *Q*(*i*) and the matched path *P*(*j, S*); *δ_D _*denotes the penalty for a gap. Note that *j *could be any vertex that is connected to vertex *m*.

### Application of the branch and bound method

The branch and bound (BB) method was initially proposed by Land [25] for the purpose of finding optimal solutions to various optimization problems, especially the discrete and combinatorial optimization problems. The objective of BB is to screen out a subset of candidate solutions by pruning a number of useless solutions. For a given candidate set *S*, a splitting procedure is utilized to divide *S *into some subsets. Another procedure is used to estimate the upper bound or lower bound of a candidate solution. The main idea of BB is to exclude those candidates whose upper bound is below the lower bound of other candidates during finding the maximum solution in *S*. Therefore, the essential task of BB is to define a proper estimator for measuring the lower and the upper bounds.

In our problem, we observed a sharp decrease in speed during DP processing when the query string reaches 10 amino acids. Therefore, we intend to apply BB to the set of candidate paths so as to optimize the candidate set by excluding some useless paths during the DP process.

### Scoring for paths and patches

The best alignment to a mimotope sequence is obtained by repeatedly performing the DP procedure. However, the alignment score for a mimotope will be inaccurate if paths with different lengths are evaluated in the same way. Therefore, *P*-value that is generated from the EVD using the method described in [19] is used as the scoring mechanism. The score for a patch is calculated as the sum of *P*-value scores of all alignments associated with that patch. Patches with the highest score are retained as the possible candidate epitopes.

### A modified BLOSUM62 substitution matrix

In bioinformatics and evolutionary biology, substitution matrices play a very important role in evaluating the homology of two amino acids. In our problem, mimotopes derived from a phage display library share a certain degree of homology with the epitopic region in most cases. Hence selection of substitution matrices has a great impact on the performance of alignment. Previously BLOSUM62 has been proven to be successful in detecting similarities in distance sequences, and used by default in some applications, such as BLAST [34]. Our study cannot satisfy the prerequisite of BLOSUM62 because a phage display library is biased in using four kinds of rib nucleotide, which has been discussed in [19]. However, the modified BLOSUM62 for NNK library can be used for our problem and thus is set as the default setting in our process.

## Results and Discussion

### A brief introduction to the MimoPro server

**Figure 3:**
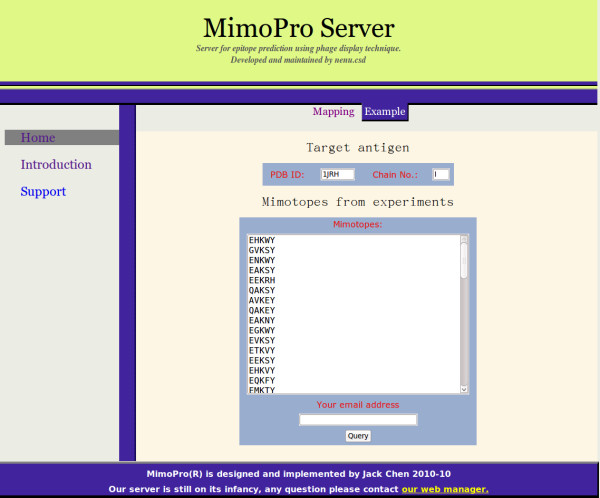
**The MimoPro server**. Users are required to specify both the four-character identifier of an antigen structure in PDB database (PDB_ID) and the identifier of the interacting chain (Chain No). Users are then required to paste the mimotopes derived from phage display experiments. An email address should be specified and the final results will be sent through this email.

The MimoPro server is currently deployed on Linux using tomcat server 6.0. It has been tested using many popular Web browsers, such as IE6, IE7, Firefox, and Opera. Queries submitted by a user are firstly stored in the server and then executed one by one in the order of FCFS (first come first serve). The processed results are displayed on a Web page, whose link is sent to the user by email. A sample demonstrating how to initiate a request is shown in Figure 3.

**Figure 4:**
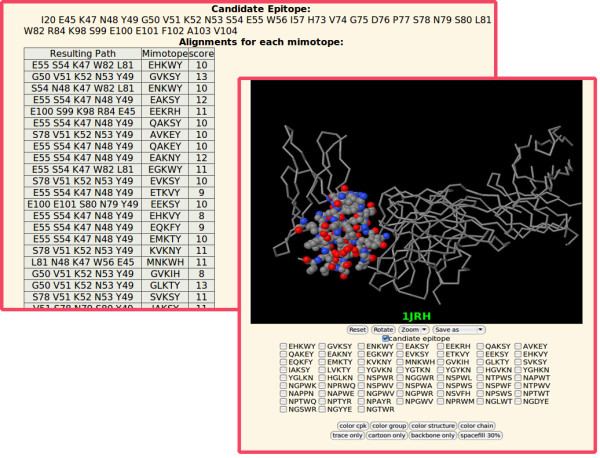
**Output of MimoPro**. The result from test case 1JRH is shown in text/tables (back) and 3D graphics (front). The candidate epitope is shown in the shape of spacefill and cpk color format by default. The display of the 3D structure can be adjusted by operations of ***Reset***, ***Rotate ***and ***Zoom***. Users can also click on the check boxes which are labeled with mimotope sequences, so that the corresponding paths on the protein surface are selected and displayed in green. Color and shape of the selected region can be altered by clicking any of the 8 buttons on the bottom. When the best view is reached, the user can save the display of the structure by choosing one of the formats provided in the droplist labeled as '***Save as***'.

The processed results displayed on the Web page include the candidate epitope and the alignments for all mimotope sequences with their *P*-value scores. These results can be viewed in either text/table or 3D graphics through Jmol [35] that can be adjusted by changing some parameters (Figure 4).

### Validation data

In practice, MimoPro requires only the crystal structure of an antigen, rather than the structure of a complex Ab-Ag. However, in order to validate the outcome of MimoPro, test cases must contain the co-crystal structure of a complex Ab-Ag since the structure of an Ab-Ag complex can tell us the exact location and construction of an epitope. Following this criterion, we collected 18 test cases from various references and each case is identified by the PDB ID of its co-crystal structure (Table 1). Test cases with references starting with 'MS' are entries in a newly released database MimoDB [36] located at http://immunet.cn/mimodb/index.html.

**Table 1:** Test cases for validation and assessment of MimoPro

PDB_ID	antibody	antigen	Mimotopes^#^	Ref
1JRH	A6, IgG1	IFNgammaR	59 × 5	[37]
1BJ1	rhuMAb	vascular endothelial growth factor	36 × 6, 3 × 5, 2 × 4	[38]
1G9M	17b	gp120	10 × 14, 1 × 12	[43]
1E6J	13B5	p24	14 × 14, 2 × 7	[43]
1N8Z	Herceptin	Her-2	5 × 12	[44]
1N8Z*	Herceptin	Her-2	8 × 12	[39]
1IQD	BO2C11	Coagulation factor VIII	27 × 12	[45]
1YY9	Cetuximab	Epidermal Growth Factor Receptor	3 × 10	[44]
2ADF	82D6A3, IgG	human von Willebrand factor (vWF)	2 × 15, 3 × 6	[46]
1ZTX	E16	West Nile Virus envelope glycoprotein(WNV E)	3 × 13, 19 × 14	[47]
3IU3	basiliximab	Interleukin-2 receptor subunit alpha	6 × 9	MS00013^$^
2GHW	80R	Spike glycoprotein	9 × 16, 11 × 15, 17 × 14, 4 × 13	MS00059^$^
2NY7	B12	Surface protein gp120	17 × 14, 1 × 10, 1 × 13	MS00056^$^
**Protein-protein interaction**				
1AVZ	Fyn	SH3 domain Nef Bovine	8 × 11, 10 × 12	[48]
1HX1	Hsc70	Bag chaperone regulator	8 × 15	[49]
1SQ0	Platelet glycoprotein Ib alpha chain	von Willebrand factor (vWF)	3 × 11	MS00465^$^
1MQ8	ICAM-1	Integrin alpha-L beta-2	1 × 14	MS01004^$^
1II4	FGFR-2	HBGF-2	30 × 7	MS01105/MS01110/MS01115^$^

^# ^Number of peptides x peptide length

* 1N8Z* shares the same crystal complex with 1N8Z in PDB database.

^$ ^These cases are entries in a newly released database MimoDB [36] located at http://immunet.cn/mimodb/index.html.

The affinity-selected peptides in cases 1JRH and 1BJ1 are derived from point mutation of the original epitopic region of the antigen [37] and of the CDR region of the antibody [38], respectively. These two cases are deemed as simple cases and thus used for validating the viability of MimoPro. The next 11 tests are real cases, in which affinity-selected peptides are mimotopes screened from the phage display libraries with their corresponding antibodies. These cases are used for mapping the mimotopes back to the source antigens so as to locate the native epitopes. The last five cases are used for mapping the affinity-selected peptides to the binding regions of interacting proteins in protein-protein interactions.

Case 1N8Z* shares the same Ab-Ag complex structure with case 1N8Z but is different in mimotopes used [39]. Mimotopes in 1YY9 include one target-unrelated peptide partly binding to the plastic plate in the phage display experiment rather than the corresponding antibody [40]. Therefore, that peptide is removed from the mimotopes of 1YY9 to avoid possible misunderstanding. Locations of native epitopes are derived from conformational epitope database (CED) [41] for those cases of Ab-Ag interaction, including 1JRH, 1G9M, 1E6J, 1N8Z, 1N8Z*, 1IQD, 1YY9, 2ADF, and 1ZTX. For other cases (3IU3, 2GHW, 2NY7, 1AVZ, 1HX1, 1SQ0, 1MQ8, and 1II4), the binding interfaces are inferred from the Contact Map Analysis (CMA) [42].

### Indicators and results

Methods of epitope prediction based on mimotope mapping can be roughly classified into two major categories: methods based on statistical features of epitopes and methods based on graph search. MimoPro belongs to the second category. Comparisons in previous studies have shown that methods based on graph search performed better than statistics-based methods on average [19]. In our study, therefore, the performance of MimoPro is only compared with that of other graph-search based methods, such as Pep-3D-Search and PepSurf. Pep-3D-Search is written in VB.net whereas PepSurf is implemented using C++. Both are freely accessible for academic use.

To evaluate the performance of MimoPro on a comparable ground with that of both PepSurf and Pep-3D-Search, we adopt the following three commonly used indictors: sensitivity (*Se*), precision (*Pr*), and Matthews correlation coefficient (*MCC*) [20]. They are defined as(5)(6)(7)

In these expressions, *TP *is the number of true positives; *FN *is the number of false negatives; *FP *is the number of false positives; *TN *is the number of true negatives.

In our study, *TP *is the number of predicted epitopic amino acids proven to be the true epitopic amino acids. *FP *is the number of predicted epitopic amino acids proven not to be the true epitopic amino acids. *TN *is the predicted non-epitopic amino acids proven not to be the true epitopic amino acids. *FN *is the number of predicted non-epitopic amino acids proven to be the true epitopic amino acids. We use *PE *to denote the number of all predicted epitopic amino acids (the sum of *TP *and *FP*).

The mapping results of all 18 test cases using MimoPro, Pep-3D-Search, and PepSurf are listed in Table 2. Note that results from both Pep-3D-Search and PepSurf are obtained using their default parameters. All these tests were conducted using the same Intel Core 2 Duo processor.

**Table 2:** Test results from MimoPro, Pep-3D-Search and PepSurf

PDB_ID	MimoPro				Pep-3D-Search				PepSurf			
	*TP*/*PE*	*Se*	*Pr*	*MCC*	*TP*/*PE*	*Se*	*Pr*	*MCC*	*TP*/*PE*	*Se*	*Pr*	*MCC*
1JRH	20/31	0.952	0.645	0.358	17/43	0.810	0.395	0.217	20/29	0.952	0.690	0.372
1BJ1	15/36	0.882	0.417	0.164	11/36	0.647	0.306	0.113	3/17	0.176	0.176	0.032
1G9M	9/50	0.600	0.180	0.058	11/43	0.733	0.256	0.080	12/40	0.800	0.300	0.092
1E6J	11/42	1.000	0.262	0.117	11/32	1.000	0.344	0.134	11/22	1.000	0.500	0.162
1N8Z	18/38	0.900	0.474	0.118	19/37	0.950	0.514	0.127	6/11	0.300	0.545	0.072
1N8Z*	18/38	0.900	0.474	0.118	20/37	1.000	0.541	0.133	0/22	0.000	0.000	0.000
1IQD	9/39	0.562	0.231	0.089	4/47	0.250	0.085	0.000	8/30	0.500	0.267	0.094
1YY9	0/43	0.000	0.000	0.000	7/24	0.500	0.292	0.056	1/8	0.071	0.125	0.012
2ADF	12/35	0.800	0.343	0.141	12/41	0.800	0.293	0.128	10/18	0.667	0.556	0.167
1ZTX	14/39	0.875	0.359	0.203	6/34	0.375	0.176	0.018	5/21	0.312	0.238	0.052
3IU3	4/24	0.143	0.167	0.017	0/13	0.000	0.000	0.000	6/26	0.214	0.231	0.045
2GHW	13/37	0.448	0.351	0.119	0/37	0.000	0.000	0.000	10/26	0.345	0.385	0.110
2NY7	3/30	0.115	0.100	0.007	3/35	0.115	0.086	0.001	6/39	0.231	0.154	0.031
1AVZ	11/32	0.688	0.344	0.150	10/31	0.625	0.323	0.133	11/18	0.688	0.611	0.217
1HX1	16/38	0.667	0.421	0.195	16/36	0.667	0.444	0.206	6/23	0.250	0.261	0.032
1SQ0	8/34	0.296	0.235	0.057	6/26	0.222	0.231	0.046	1/15	0.037	0.067	0.000
1MQ8	7/30	0.412	0.233	0.072	2/29	0.118	0.069	0.000	0/12	0.000	0.000	0.000
1II4	23/41	0.622	0.561	0.249	23/44	0.622	0.523	0.233	12/22	0.324	0.545	0.163
**Average**		**0.603**	**0.322**	**0.124**		**0.524**	**0.271**	**0.082**		**0.382**	**0.314**	**0.089**

*TP*: number of true positive; *PE*: number of predicted epitope; *Se*: sensitivity; *Pr*: precision; *MCC*: Matthews correlation coefficient.

### Feasibility of MimoPro

For the first validation case 1JRH, the known mimotopes mutated from the epitopic region of the source antigen are E45_V46_K47_N48_Y49, Y49_G50_V51_K52_N53, and N53_S54_E55_W56_I57, with a high similarity to the native epitope. Among these mimotope sequences, YGVKN is identical to the native epitopic segment Y49_G50_V51_K52_N53. MimoPro successfully located this epitope on the antigen surface. The resultant patch consists of 31 residues, among which 20 residues are projected to the genuine region of the native epitope. The best matched path on the resultant patch to mimotope sequence YGVKN is Y49_G50_V51_k52_N53, identical to the native epitopic segment. PepSurf and Pep-3D-Search predicted 20 and 17 hits for 1JRH respectively (Table 2). Generally for this validation case, MimoPro and PepSurf have a similar sensitivity, precision and MCC, both being clearly better than Pep-3D-Search.

For the second validation case 1BJ1 where affinity-selected peptides from the peptide library were generated from the randomly mutated CDR region of the antibody, the candidate epitope identified by MimoPro contains 36 residues, in which 15 correlate to the native epitope region that consists of 17 amino acids. PepSurf and Pep-3D-Search predicted 11 and 3 hits for 1BJ1 respectively (Table 2), which puts MimoPro as the absolute best performer among these three in this case.

The next 11 real cases produced mixed outcomes, which indicate that no one dominates over others in all circumstances but each has its advantage in particular cases (Table 2). It is worth mentioning that all mimotopes in these cases, derived from random peptide libraries, show a low sequence homology with the genuine epitopes, and most of these epitopes are discontinuously distributed in separate segments. In general, MimoPro performed better in 1ZTX, 2ADF and 2GHW but slightly worse in 1G9M and 1E6J than both PepSurf and Pep-3D-Search did. In 1IQD that consists of 16 amino acids, no superb result was produced by all three methods. However, MimoPro performed slightly better than the other two because it identified more than half of the genuine epitope.

In 1YY9, MimoPro failed in producing any useful result. This is an extremely hard case where the 14 amino acids forming the epitope include three consecutive segments (Q408_H409, V417_S418, and K465_I466_I467_S468) and six isolated amino acids (R353, Q384, Q438, S440, K443, and N473). As a result, outcomes from both PepSurf and Pep-3D-Search for 1YY9 were not good either.

PepSurf was the best performer in cases 1G9M, 1E6J, 3IU3 and 2NY7 but the worst in 1N8Z, 2ADF and 1ZTX, and even failed in 1N8Z*. On the other hand, Pep-3D-Search produced the best results in both 1N8Z and 1N8Z*, but was rated the last in 1IQD and 2NY7, and even failed in both 3IU3 and 2GHW.

For the last five cases, MimoPro was the best performer in 1SQ0, 1MQ8 and 1II4, and similar to both/either PepSurf and/or Pep-3D-Search in 1AVZ and 1HX1 in predicting the epitopic region of protein-protein interaction. This puts MimoPro as the most effective tool among the three in predicting the epitopic regions of protein-protein interactions.

On average from the 18 test cases, MimoPro achieved the best performance in sensitivity, precision and *MCC*, compared with both PepSurf and Pep-3D-Search (Table 2). However, this may still be insufficient to specify which method is the best choice because statistics from this small test set can be greatly influenced by a few 'worse' cases. Therefore, more tests using more openly accessible databases for mimotope-based epitope prediction are required before a conclusion on performances of various methods, including MimoPro, can be made.

### Impact of compactness factor (*CF*)

In MimoPro, graph generation from a patch uses an ADT regulated by a uniform *CF*. This means that individual graphs contain a relatively certain number of edges connecting vertices. Traditionally, graphs delineated using an FDT contain different numbers of edges. To assess the impact of *CF *on the performance of MimoPro, tests on graphs resulted from using both an ADT guided by a *CF *of 0.73 ± 0.06 and an FDT of 6.5 Å have been conducted against all 18 cases used earlier. The results are shown in Table 3.

**Table 3:** Assessment on impact of compactness factor (*CF*)

PDB_ID	FDT (6.5 Å)			*CF *(0.73 ± 0.06)		
	Average CF*	Execution time (s)	*TP*/*PE*	Average *CF*	Execusion time (s)	*TP*/*PE*
1JRH	0.825	9	19/39	0.729	8	20/31
1BJ1	0.754	22	14/36	0.722	13	15/36
1G9M	0.79	402	9/50	0.719	128	9/50
1E6J	0.794	226	8/39	0.734	121	11/42
1N8Z	0.788	3147	17/37	0.713	234	18/38
1N8Z*	0.788	4845	17/37	0.713	359	18/38
1IQD	0.754	302	9/39	0.739	124	9/39
1YY9	0.822	897	0/43	0.720	128	0/43
2ADF	0.777	28	0/31	0.732	11	12/35
1ZTX	0.730	44	14/39	0.738	62	14/39
3IU3	0.828	14	11/31	0.773	13	4/24
2GHW	0.972	2466	13/37	0.756	433	13/37
2NY7	0.872	735	2/43	0.783	297	3/30
1AVZ	0.867	209	0/43	0.742	40	11/32
1HX1	0.861	374	10/35	0.729	62	16/38
1SQ0	0.65	12	6/32	0.75	18	8/34
1MQ8	0.73	2	7/30	0.75	4	7/30
1II4	0.74	31	23/41	0.76	35	23/41

* Average *CF *is calculated as the mean value of *CF*s from all the graphs generated from each surface patch.

In ten of the 18 cases, MimoPro produced more predicted hits on patches linked to graphs using an ADT regulated by a *CF *of 0.73 ± 0.06 than that on corresponding patches linked to graphs using an FDT of 6.5 Å, except 3IU3 being opposite. There is no difference in predicted hit between the two approaches in other seven cases (1G9M, 1IQD 1YY9, 1ZTX, 2GHW, 1MQ8, and 1II4). This indicates that patches linked to graphs generated using an ADT allow MimoPro to produce better or no worse results than those generated using an FDT do. Note that both approaches still failed in returning any useful hit in case 1YY9.

*CF *is originally introduced to regulate the complexity of the searching algorithm. In most test cases, processing of MimoPro on a *CF*-regulated graph is much faster than that on a graph generated by an FDT of 6.5 Å. The most improved cases are 1N8Z and 1N8Z* that show the processing time was dramatically reduced from 3147 and 4845 seconds in circumstances of using this FDT to 234 and 359 seconds respectively in circumstances of using the ADT regulated by the specified *CF *(Table 3). This is because the source antigen of either 1N8Z or 1N8Z* contains many compact regions and thus a patch covering such region includes significantly more residues than that covering a loose region does. More residues (vertices) imply more time in processing.

Using the same principle, we can calculate the *CF *of each graph defined using an FDT of 6.5 Å (Table 3). Comparing the paired *CF*s of all cases, except 1ZTX, 1SQ0, 1MQ8 and 1II4, the *CF *of each graph defined using the FDT is greater than that of its corresponding graph generated using the ADT regulated by a *CF *of 0.73 ± 0.06. This means that the former contains more vertices than the latter does, and hence the former requires more processing time than the latter does. Similarly, it is easier to understand why in all exceptional cases the former performed faster than the latter. This is because the former has a lower *CF *than the latter has.

In summary, firstly, a graph with a higher *CF *means a higher computing cost but that with a lower *CF *may not cover sufficient number of residues required for mapping a mimotope sequence. Therefore, choosing an appropriate *CF *based on empirical data is vital in achieving a satisfactory performance in using MimoPro for epitope prediction. Secondly, MimoPro on graphs regulated by an appropriate *CF *is more sensitive in detecting epitopic amino acids in most cases than both PepSurf and Pep-3D-Search. Thirdly, MimoPro on graphs regulated by an appropriate *CF *is more efficient in real applications of epitope prediction because in most cases a result should be produced within 2 minutes, with a maximum limit of 6 minutes for a very difficult case. In this regard, however, PepSurf requires a few hours to process a single peptide sequence if the peptide contains 14 amino acids [19]; Pep-3D-Search is not better either because generating the empirical distribution to get the *P*-value alone takes 10 minutes [20].

### Effects of other parameters

Our method contains two major steps: generation of graph from each surface patch, and selection of the graph with the highest score by aligning every mimotope sequence to each graph using a complete searching algorithm. To generate a graph from a surface patch, the position of a vertex should be specified first by choosing one of the three centers at ***C_α_***, ***C_β _***or AHA. In the second step, the scoring of each path depends on the selection of substitution matrices and penalties for gaps. In our study, we assessed the impacts of these parameters on the performance of MimoPro. Results are provided in Additional file 1.

## Conclusions

In this study, we proposed a new graph-based mapping method for epitope prediction using affinity-selected peptides derived from phage display experiments. The core of our method is a searching algorithm operated on a series of overlapping patches on the surface of a protein. These patches are then transformed to graphs using an ADT regulated by *CF*, a novel parameter proposed in this study. These graphs contain a certain number of vertices, which can ensure that searching for each graph is more efficient. This is vastly different from traditional graph-based searching methods that adopt an FDT to define graphs that vary in number of vertices. Searching a graph with a large number of vertices is always slow.

Compared with Pep-3D-Search and PepSurf, two leading graph-based search tools, testing results from MimoPro, the Web-based implementation of our proposed method, have shown that in most cases MimoPro performed equally to or better than both Pep-3D-Search and PepSurf did. On average from 18 test cases, the performance of MimoPro indicated by sensitivity, precision and *MCC *is better than that of both Pep-3D-Search and PepSurf in epitope prediction. This implies that MimoPro is a viable alternative to, if not the preferred choice, both PepSurf and Pep-3D-Search for epitope prediction in the same kind.

What makes MimoPro more promising over both PepSurf and Pep-3D-Search is that searching over well constructed graphs using an ADT regulated by an appropriate *CF *is significantly and consistently faster than that of both PepSurf and Pep-3D-Search. This is mainly because such regulated graphs contain a certain number of vertices, which can guarantee that searching for each graph is faster. This further proves that our original concept for improving the search algorism is correct, feasible, and practically useful.

However, for extremely difficult cases where amino acids forming the epitope include both consecutive segments and isolated amino acids, such as 1YY9, MimoPro failed in producing any useful mappings. This indicates where our method can be further improved. Potentially improvement could be made in the following ways. Firstly, the searching algorithm itself could be modified so that the highly rated patches are searched first to make searching more efficient. Secondly, a more appropriate substitution matrix according to a specific application should be adopted so that graph rating is more meaningful to such application. In addition, a refinement model could be introduced to eliminate those insignificant amino acids on a patch so as to accelerate the processing.

## Availability and requirements

•**Project Name: **MimoPro

•**Project Homepage: **http://informatics.nenu.edu.cn/MimoPro/

•**Operating System: **Platform independent

•**Programming language: **C++

•**Other requirements: **JRE 5.0 or higher

•**License: **GNU GPL

•**Any restrictions to use by non-academics: **license needed for commercial use

## List of abbreviations used

ADT: adaptable distance threshold; ASA: accessible surface area; BB: branch and bound; *CF*: compactness factor; DP: dynamic programming; EVD: extreme value distribution; FDT: fixed distance threshold; *MCC*: Matthews correlation coefficient; PDB: protein data bank; *Pr*: precision; RSA: relative solvent accessibility; *Se*: sensitivity

## Authors' contributions

WHC conceived the idea of this *CF*-regulated graph search algorithm and was in charge of the MimoPro implementation. He also drafted the first version of the manuscript. PPS and ZQM optimized the algorithm and participated in the development and validation of the Web server. YL and YXH designed experiments, gathered test data, and were in charge of the experiments. YXH supervised the progress of the whole project and critically checked the first draft. WWG was in charge of the whole process of final revision. Working with other authors, particularly WHC, he restructured the paper and rationalized Background, Results and Discussion, and Conclusions, based on the comments received from the reviewers and editors. All authors have read and approved the final manuscript.

## Supplementary Material

Additional file 1 **Supplementary experiment results**. Additional file 1 is a single PDF file which includes tables 1 to 3 of supplementary experiment results. Click here for file
