# Supplementary material for: MimoPro: a more efficient Web-based tool for epitope prediction using phage display libraries
Source: BMC Bioinformatics. 2011 May 25;12:199. doi: 10.1186/1471-2105-12-199 (PMC3124435; doi:10.1186/1471-2105-12-199)
Supplement: Additional file 1 — Supplementary experiment results. Additional file 1 is a single PDF file which includes tables 1 to 3 of supplementary experiment results. [file 1471-2105-12-199-S1.PDF]

Additional files 1

S1 Optimization of compactness factor (*CF*)

No.	1JRH	1BJ1	1G9M	1E6J	1N8Z	1N8Z*	1IQD	1YY9	2ADF	1ZTX	3IU3	2GHW	2NY7	1AVZ	1HX1	1SQ0	1MQ8	1II4	
CF(0.70)																			
TP/PE	19/39	15/36	9/50	11/39	17/37	17/37	3/30	0/50	12/35	14/39	4/24	13/37	3/30	11/32	11/35	6/32	7/30	23/41	
Se	0.905	0.882	0.600	1.000	0.850	0.850	0.188	0.000	0.800	0.875	0.143	0.448	0.115	0.688	0.458	0.222	0.412	0.622	0.559
Pr	0.487	0.417	0.180	0.282	0.459	0.459	0.100	0.000	0.343	0.359	0.167	0.351	0.100	0.344	0.314	0.188	0.233	0.561	0.297
MCC	0.287	0.164	0.058	0.121	0.113	0.113	0.000	0.000	0.141	0.203	0.017	0.119	0.007	0.150	0.093	0.031	0.072	0.249	0.107
CF(0.71)																			
TP/PE	19/39	15/36	9/50	8/45	18/38	17/37	3/30	0/50	12/35	14/39	4/24	13/37	3/30	11/32	16/38	5/35	7/30	23/41	
Se	0.905	0.882	0.600	0.727	0.900	0.850	0.188	0.000	0.800	0.875	0.143	0.448	0.115	0.688	0.667	0.185	0.412	0.622	0.556
Pr	0.487	0.417	0.180	0.178	0.474	0.459	0.100	0.000	0.343	0.359	0.167	0.351	0.100	0.344	0.421	0.143	0.233	0.561	0.295
MCC	0.287	0.164	0.058	0.075	0.118	0.113	0.000	0.000	0.141	0.203	0.017	0.119	0.007	0.150	0.195	0.009	0.072	0.249	0.109
CF(0.72)																			
TP/PE	20/38	15/36	9/50	8/45	18/38	17/37	13/43	0/43	12/35	14/39	4/24	13/37	3/30	11/32	16/38	8/34	7/30	23/41	
Se	0.952	0.882	0.600	0.727	0.900	0.850	0.812	0.000	0.800	0.875	0.143	0.448	0.115	0.688	0.667	0.296	0.412	0.622	0.599
Pr	0.526	0.417	0.180	0.178	0.474	0.459	0.302	0.000	0.343	0.359	0.167	0.351	0.100	0.344	0.421	0.235	0.233	0.561	0.314
MCC	0.316	0.164	0.058	0.075	0.118	0.113	0.146	0.000	0.141	0.203	0.017	0.119	0.007	0.150	0.195	0.057	0.072	0.249	0.122
CF(0.73)																			
TP/PE	20/31	15/36	9/50	11/42	18/38	18/38	9/39	0/43	12/35	14/39	4/24	13/37	3/30	11/32	16/38	8/34	7/30	23/41	
Se	0.952	0.882	0.600	1.000	0.900	0.900	0.562	0.000	0.800	0.875	0.143	0.448	0.115	0.688	0.667	0.296	0.412	0.622	0.603
Pr	0.645	0.417	0.180	0.262	0.474	0.474	0.231	0.000	0.343	0.359	0.167	0.351	0.100	0.344	0.421	0.235	0.233	0.561	0.322
MCC	0.358	0.164	0.058	0.117	0.118	0.118	0.089	0.000	0.141	0.203	0.017	0.119	0.007	0.150	0.195	0.057	0.072	0.249	0.124
CF(0.74)																			
TP/PE	20/38	15/36	9/50	11/42	18/38	18/38	9/39	0/43	12/35	14/39	4/24	13/37	3/30	11/32	16/38	8/34	7/30	23/41	
Se	0.952	0.882	0.600	1.000	0.900	0.900	0.562	0.000	0.800	0.875	0.143	0.448	0.115	0.688	0.667	0.296	0.412	0.622	0.603
Pr	0.526	0.417	0.180	0.262	0.474	0.474	0.231	0.000	0.343	0.359	0.167	0.351	0.100	0.344	0.421	0.235	0.233	0.561	0.315
MCC	0.316	0.164	0.058	0.117	0.118	0.118	0.089	0.000	0.141	0.203	0.017	0.119	0.007	0.150	0.195	0.057	0.072	0.249	0.121
CF(0.75)																			
TP/PE	19/38	15/36	8/50	11/42	18/38	18/38	9/39	0/43	13/24	14/39	4/24	0/42	3/30	11/32	16/38	6/32	7/30	23/41	
Se	0.905	0.882	0.533	1.000	0.900	0.900	0.562	0.000	0.867	0.875	0.143	0.000	0.115	0.688	0.667	0.222	0.412	0.622	0.572
Pr	0.500	0.417	0.160	0.262	0.474	0.474	0.231	0.000	0.542	0.359	0.167	0.000	0.100	0.344	0.421	0.188	0.233	0.561	0.302
MCC	0.292	0.164	0.050	0.117	0.118	0.118	0.089	0.000	0.190	0.203	0.017	0.000	0.007	0.150	0.195	0.031	0.072	0.249	0.109

We assessed the performance of MimoPro by changing *CF* from 0.70 to 0.75. On average, sensitivity, precision and *MCC* are relatively stable in this range. The best result is from a *CF* of 0.73 considering all three indicators. Therefore, it is set as the default value for *CF* in MimoPro.

S2 Experimental results of using different strategies for centering residues

PDB_ID	C_β (0.73)				C_α (0.71)			
	**TP/PE**	**Se**	**Pr**	**MCC**	**TP/PE**	**Se**	**Pr**	**MCC**
1JRH	20/31	0.952	0.645	0.358	19/39	0.90	0.487	0.287
1BJ1	15/36	0.882	0.417	0.164	15/36	0.88	0.417	0.164
1G9M	9/50	0.600	0.180	0.058	9/50	0.60	0.180	0.058
1E6J	11/42	1.000	0.262	0.117	11/39	1.00	0.282	0.121
1N8Z	18/38	0.900	0.474	0.118	17/37	0.85	0.459	0.113
1N8Z*	18/38	0.900	0.474	0.118	17/37	0.85	0.459	0.113
1IQD	9/39	0.562	0.231	0.089	3/30	0.18	0.100	0.000
1YY9	0/43	0.000	0.000	0.000	0/50	0.00	0.000	0.000
2ADF	12/35	0.800	0.343	0.141	12/35	0.80	0.343	0.141
1ZTX	14/39	0.875	0.359	0.203	14/39	0.87	0.359	0.203
3IU3	4/24	0.143	0.167	0.017	19/40	0.67	0.475	0.188
2GHW	13/37	0.448	0.351	0.119	11/32	0.37	0.344	0.104
2NY7	3/30	0.115	0.100	0.007	2/41	0.07	0.049	0.000
1AVZ	11/32	0.688	0.344	0.150	11/32	0.68	0.344	0.150
1SQ0	16/38	0.667	0.421	0.195	11/35	0.45	0.314	0.093
1MQ8	8/34	0.296	0.235	0.057	6/28	0.22	0.214	0.041
1II4	7/30	0.412	0.233	0.072	5/45	0.29	0.111	0.011
1HX1	23/41	0.622	0.561	0.249	25/33	0.67	0.758	0.331
Average		0.603	0.322	0.124		0.57	0.316	0.116

Experiments are designed for choosing different atoms as the center of an amino acid. Results listed were from 12 test cases by increasing *CF* from 0.70 to 0.75. The best results of MimoPro using $\boldsymbol{C_\beta}$ atom as center of a residue are reached with a *CF* of 0.73 whereas that using $\boldsymbol{C_\alpha}$ atom as the center are obtained with a *CF* of 0.71. On average, MimoPro using $\boldsymbol{C_\beta}$ atom as the center has a better performance in sensitivity, precision and *MCC* over the other.

S3 Impacts of gap penalty

PDBID	Penalty -0.5				Penalty -1.5				Penalty -2.5				Penalty -3.5			
	TP/PE	Se	Pr	MCC	TP/PE	Se	Pr	MCC	TP/PE	Se	Pr	MCC	TP/PE	Se	Pr	MCC
1JRH	19/39	0.90	0.48	0.28	19/39	0.90	0.48	0.28	19/39	0.90	0.48	0.28	20/31	0.95	0.64	0.35
1BJ1	15/36	0.88	0.41	0.16	15/36	0.88	0.41	0.16	15/36	0.88	0.41	0.16	15/36	0.88	0.41	0.16
1G9M	9/50	0.60	0.18	0.05	9/50	0.60	0.18	0.05	9/50	0.60	0.18	0.05	9/50	0.60	0.18	0.05
1E6J	11/39	1.00	0.28	0.12	11/39	1.00	0.28	0.12	11/39	1.00	0.28	0.12	11/42	1.00	0.26	0.11
1N8Z	17/37	0.85	0.45	0.11	17/37	0.85	0.45	0.11	17/37	0.85	0.45	0.11	18/38	0.90	0.47	0.11
1N8Z*	17/37	0.85	0.45	0.11	17/37	0.85	0.45	0.11	17/37	0.85	0.45	0.11	18/38	0.90	0.47	0.11
1IQD	3/30	0.18	0.10	0.00	3/30	0.18	0.10	0.00	3/30	0.18	0.10	0.00	9/39	0.56	0.23	0.08
1YY9	0/50	0.00	0.00	0.00	0/50	0.00	0.00	0.00	0/50	0.00	0.00	0.00	0/43	0.00	0.00	0.00
2ADF	12/35	0.80	0.34	0.14	12/35	0.80	0.34	0.14	12/35	0.80	0.34	0.14	12/35	0.80	0.34	0.14
1ZTX	14/39	0.87	0.35	0.20	14/39	0.87	0.35	0.20	14/39	0.87	0.35	0.20	14/39	0.87	0.35	0.20
3IU3	12/34	0.42	0.35	0.11	4/24	0.14	0.16	0.01	4/24	0.14	0.16	0.01	4/24	0.14	0.16	0.01
2GHW	0/41	0.00	0.00	0.00	13/37	0.44	0.35	0.11	13/37	0.44	0.35	0.11	13/37	0.44	0.35	0.11
2NY7	3/30	0.11	0.10	0.00	3/30	0.11	0.10	0.00	3/30	0.11	0.10	0.00	3/30	0.11	0.10	0.00
1AVZ	11/32	0.68	0.34	0.15	11/32	0.68	0.34	0.15	11/32	0.68	0.34	0.15	11/32	0.68	0.34	0.15
1HX1	11/35	0.45	0.31	0.09	11/35	0.45	0.31	0.09	11/35	0.45	0.31	0.09	16/38	0.66	0.42	0.19
1SQ0	0/28	0.00	0.00	0.00	8/34	0.29	0.23	0.05	8/34	0.29	0.23	0.05	8/34	0.29	0.23	0.05
1MQ8	7/39	0.41	0.17	0.05	7/30	0.41	0.23	0.07	7/30	0.41	0.23	0.07	7/30	0.41	0.23	0.07
1II4	23/41	0.62	0.56	0.24	22/41	0.62	0.56	0.24	23/41	0.62	0.56	0.24	23/41	0.62	0.56	0.24
		0.53	0.27	0.09		0.56	0.30	0.10		0.56	0.30	0.10		0.60	0.32	0.12

The impact of gap penalty was assessed by increasing the gap penalty from -0.5 to -3.5. In general, performance keeps with an increase in gap penalty. This indicates that a gap penalty of -3.5 is mostly appropriate to our searching algorithm.
